# A Learned Judge

**Published:** 1891-04

**Authors:** 


### A LEARNED JUDGE.

THE *Medical Standard* for October publishes the decision of Chief Justice Thayer, of Oregon, in a recent malpractice suit, of which the following is a brief summary: He holds that the practice of the courts, in allowing juries to determine these cases, is unjust to the medical profession, because it encourages the institution of suits against physicians. The surgeon cannot always be expected to achieve the success he desires, for the reason that causes for which he cannot be responsible may interfere. The average juror is unfitted to decide such cases, and the trial court should never allow them to be submitted to the jury unless the plaintiff has shown by proof that the defendant is guilty of the charge. People who devote their lives to relieving the suffering of mankind should be encouraged, and the way suits for malpractice are often conducted has the opposite effect, and is apt to leave the performance of many important duties to reckless and irresponsible empirics.

The decision of Chief Justice Thayer, of Oregon, is rational and just. The profession has long suffered from the injustice of jurors, whose prejudices and sympathies have given ruinous and unjust verdicts against physicians and surgeons. Men devote their lives to the study of their profession, render eminent service to their patients, and then are compelled to pay heavy damages because nature was too weak to respond to remedies. For quacks we have no sympathy. We would have them suffer just penalties for trifling, in their ignorance and unskilfulness, with the bodies and lives of men. It is the surgeon, in particular, who stands most in need of the protection of such a rule as the Oregon judge lays down. The most skilful surgeon cannot be an insurer against the resistance of nature to his treatment.

The sympathy of the jury is usually with the plaintiff in advance, and it is grossly unjust in a judge to allow a malpractice case to go to the jury, unless expert testimony justifies it. The judge should be impartial. He should be the protector of the physician and surgeon against passion and prejudice and sympathy, as he should be of the plaintiff against unskilfulness and quackery. Unless a case is made by scientific witnesses against the party sued for malpractice, the Court should refuse to allow the case to go to the jury. In commercial cases, the Court grants a non-suit, unless a *prima facie* case is made. Why a different rule when the reputation of a physician or a surgeon is at stake, and a heavy claim for damages is made?

Suits for malpractice are often brought by poor patients, who have been gratuitously treated, instigated by lawyers who take the chances of a large verdict for their large reward. They are speculative suits, which courts should not encourage. Honorable members of our profession devote years to preparation for its duties, and their studies never end. They respond to the call of the poor as readily as of the rich, and, with few exceptions, the pecuniary results of their toilsome lives is small. Their reputation is sacred to them, and, while we have no plea for the quack, we invoke the protection of the courts against the speculative persecutions and prosecutions of men who give honest and intelligent service to their patients.

